# Endodontic management of a tooth with apical overfilling and perforating external root resorption: A case report

**DOI:** 10.1002/ccr3.3406

**Published:** 2020-10-15

**Authors:** Reza Sayyad Soufdoost, Ali Jamali Ghomi, Hossein Labbaf

**Affiliations:** ^1^ Endodontics Department Faculty of Dentistry Shahed University Tehran Iran; ^2^ Prosthodontics Department Faculty of Dentistry Shahed University Tehran Iran

**Keywords:** biodentine, endodontic, external root resorption, overfilling

## Abstract

Successful non‐surgical orthograde retreatment of a tooth with external root resorption in apical third of root and overfilling material beyond the apical barrier which was diagnosed with the help of cone beam computed tomography (CBCT), was reported. Biodentine was used as the treatment of choice for obturation of resorption area.

## Graphical abstract

Overfilling management can be done by performing nonsurgical endodontic procedure. Gutta‐percha of coronal and middle third of root should be removed by mechanical instruments; however, in apical, removal should be done by hand instrument. Biodentine can be considered as the treatment of choice for filling the perforating external root resorption.

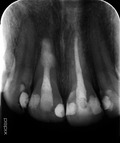

## 1 INTRODUCTION

The external root resorption (ERR) is a progressive inflammatory process in which inflammatory cells, resorbing cells (clasts), and hard tissues are involved.^1^ Cementum and predentin covering on dentin are mandatory components which preserve dental root from resorption.^2^ Major mediators of osteoclasts binding are RGD peptides that are bound to calcium crystals on mineralized surface.^3^ As the most external aspect of cementum is covered by cementoblasts, the satisfactory condition is not present for osteoclasts bounding.^3^ Also, the cemental layer has the ability to inhibit the movement of toxins.^2^ In a case of trauma, the cemental layer is lost or damaged, and the inflammatory stimulators from infected pulp can reach to the surrounding periodontal by passing through the dentinal tubules.^4^

The objective of nonsurgical treatment is to rectify the deficiencies of the original treatment.^5^ In order to negotiate to root canal system, root canal filling materials should be removed completely.^6^ The removal of root filling material is a key factor in root canal retreatment. Incomplete removal of all the previous root filling material may deactivate the chemo‐mechanical cleaning of the root canal system and prevent correcting the deficiencies associated with the original root canal.^5^, ^6^ The quality of root canal obturation was the most important factor in the success of the endodontic treatment.^5^ According to Swartz et al^7^ overextended obturation is four times more likely to fail than under obturated canals.

Cone‐beam computed tomography (CBCT) is a diagnostic imaging modality which affords clinicians the ability to accurate diagnosis, treatment planning, and follow‐up.^8^ CBCT provides a detailed three‐dimensional evaluation of teeth, maxillofacial skeletal district, and relation among anatomical structures comparing the two‐dimensional images provided by conventional intraoral periapical radiographs.^8^, ^9^ Particularly, in the case of ERR, it is important to understand whether there is a communicating lesion or it is limited in the external surface without involvement of root canal space.^9^

This case report presents orthograde endodontic retreatment of a tooth with extrusion of endodontic filling material beyond the apical region combined with incomplete obturation of the resorption area.

## 2 CASE REPORT

**FIGURE 1:**
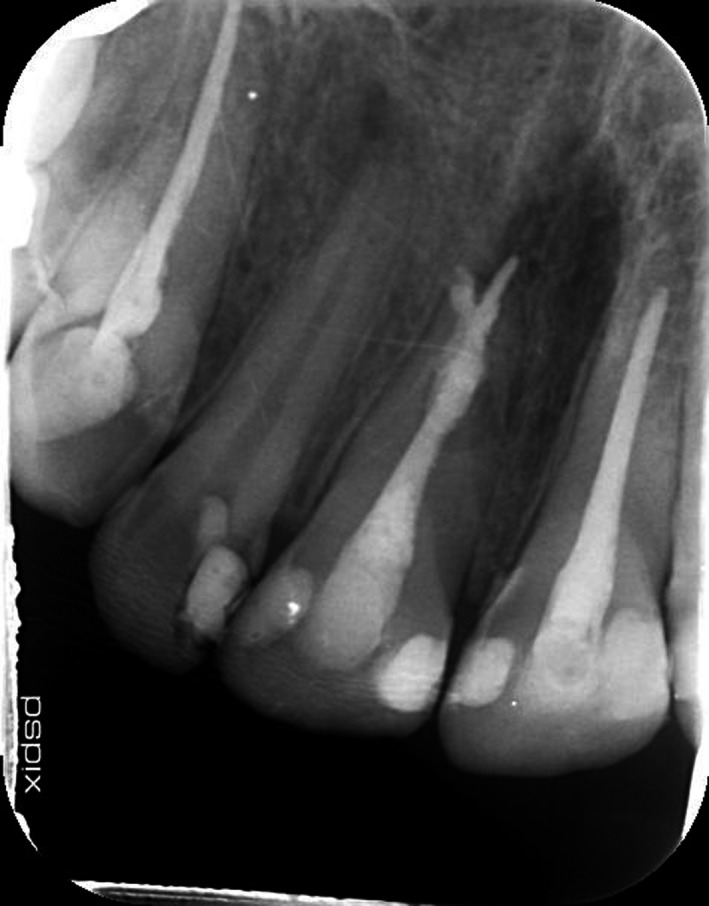
Postoperative PA of initial treatment by first dentist

**FIGURE 2:**
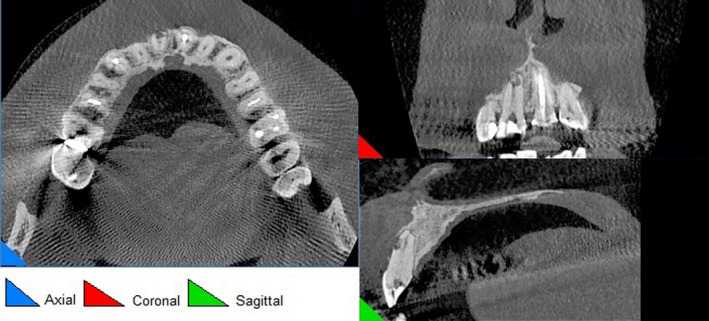
Coronal, sagittal, and axial view on CBCT

**FIGURE 3:**
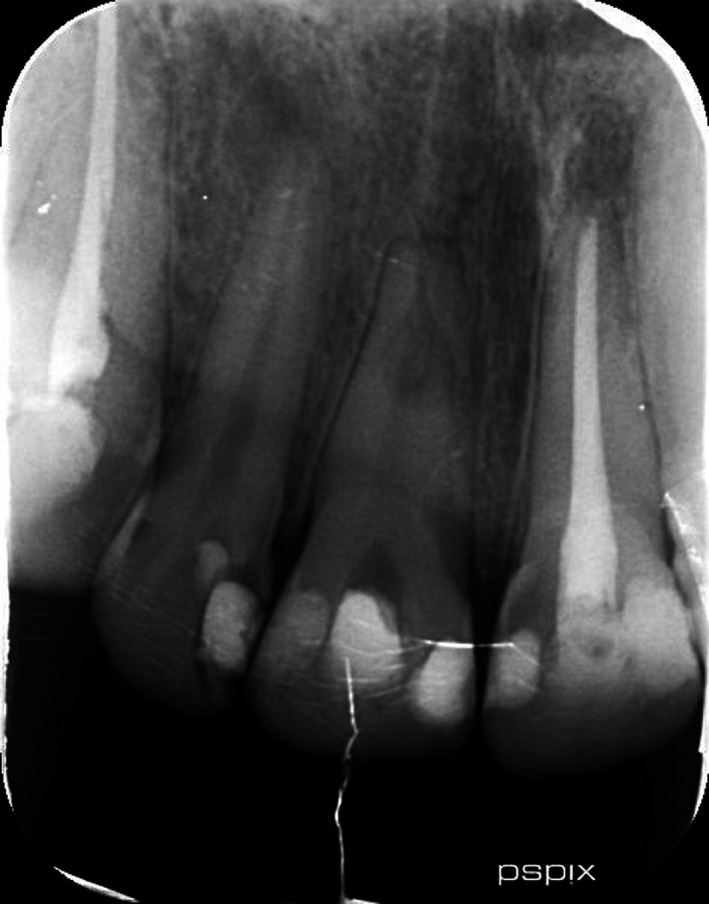
Initial PA

**FIGURE 4:**
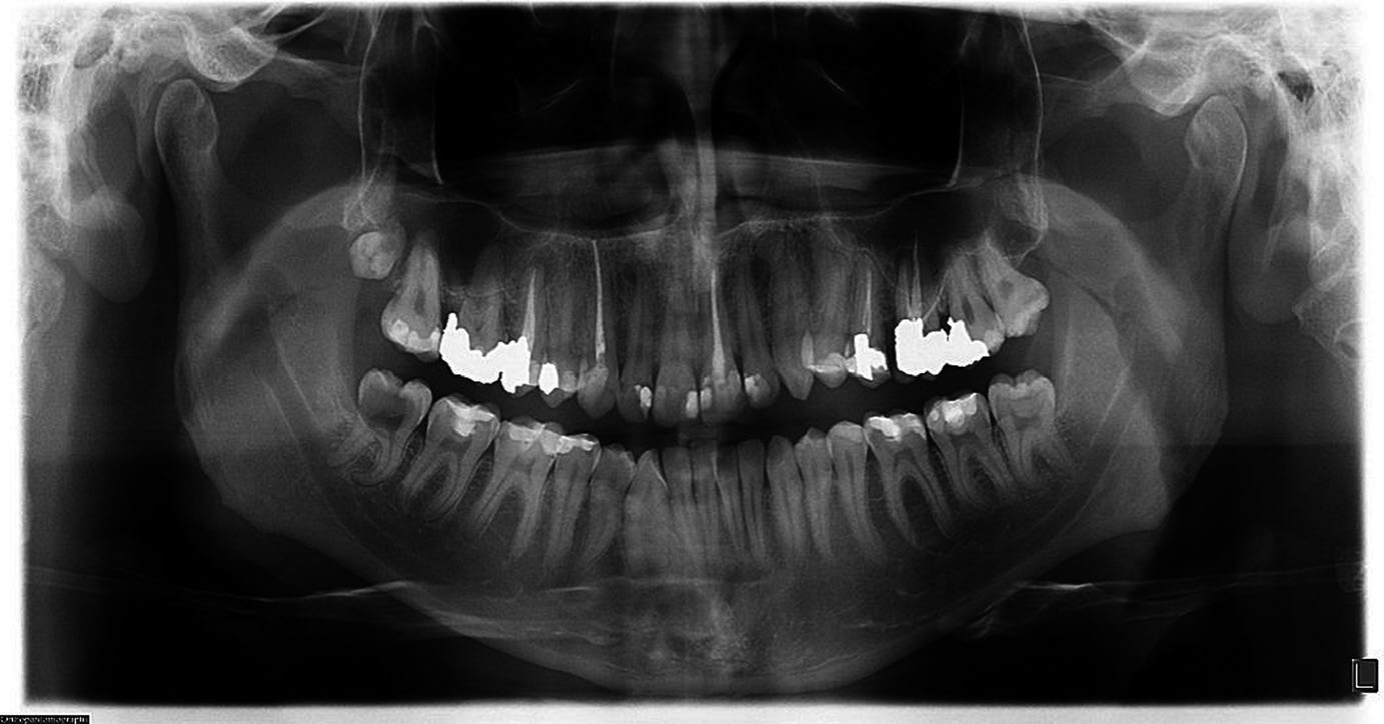
OPG

Twenty‐eight‐year‐old male patient was referred to Endodontics Department of Shahed University, with a chief complaint of pain following initial root canal therapy of tooth 11 performed by a dentist in a private office. Medical history of the patient was noncontributory. In the written referral letter, the diagnosis of internal root resorption had been made for tooth 11 by the previous dentist. Tooth 11 endodontically had been treated and obturated by gutta‐percha, but the area of resorption was not obturated and sealed appropriately based on postoperative radiograph attached to the referral letter (Figure 1). CBCT (Figure 2), initial periapical radiograph (PA) (Figure 3), and orthopantomogram (OPG) (Figure 4) had been attached to referral letter as well.

Radiographic evaluation of initial PA and OPG revealed the radiolucency around the apical third of tooth 11. Also, a remarkable gap between filling material and root's wall was evident on postoperative radiograph. On the other hand, about 2‐mm gutta‐percha was extruded beyond the confines of root canal system. Intraoral examination showed the swelling above tooth 11, weak discoloration of crown, and pain in percussion. A new periapical radiograph of tooth 11 was captured which confirmed the postoperative radiograph.

According to radiographic features, the diagnosis of external root resorption was made which contradicted the initial diagnosis, and it was confirmed by CBCT in three different plans (sagittal, coronal, and axial). Also, the CBCT showed a communicating ERR through the apical third of the root canal. It was decided to endodontically retreat tooth 11 and obturate resorption area by Biodentine (Septodont). The poor prognosis and risk of the endodontic retreatment were explained completely for patient and consent form was signed by him.

**FIGURE 5:**
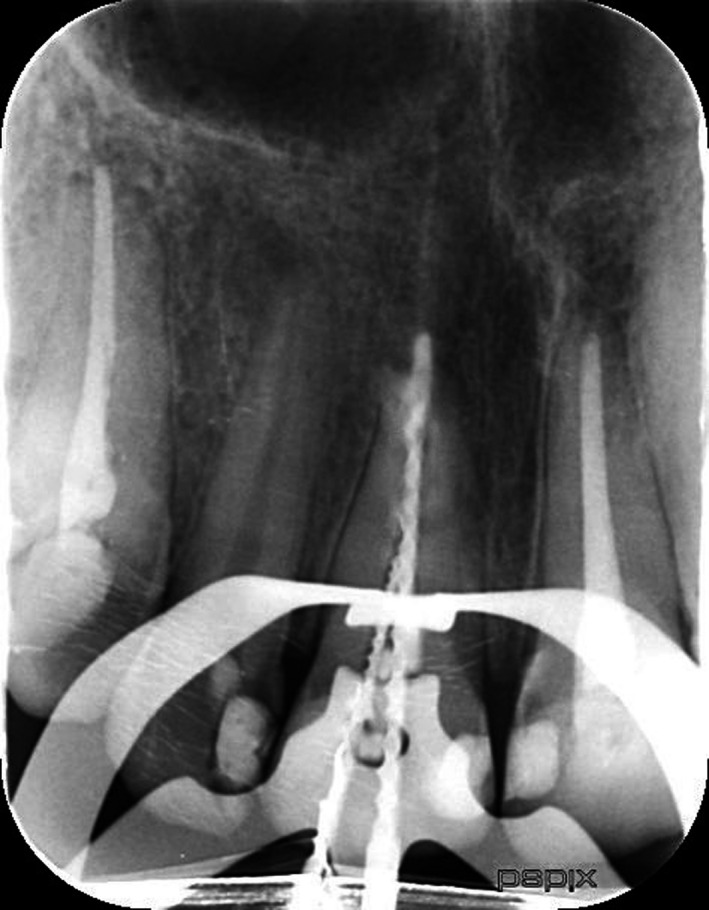
Working length PA

After anesthesia with lidocaine with 1:100 000 epinephrine (Darou Pakhsh) and isolation of tooth 11 by rubber dam, access cavity was prepared. The gutta‐percha in the coronal third of root was removed by gates glidden drill no 2 and 3 (Mani, Inc), and in middle third, the ultrasonic retreatment tip Et‐20 (size 20, 0.06 taper; no cutting blades) (Satelec Acteon Products) was used for the removal of root canal filling material. In order to remove the gutta‐percha of apical third, a novel technique was used, as K file# 40 was placed in the center of the root canal, and H file#30 wrapped clockwise around that gently as apically as possible. Once the over obturated gutta‐percha was trapped by tip of the files, with the pull‐up movement of both files, the extruded gutta‐percha was removed. Area of resorption and apical third of root canal were inspected with an operating microscope (OPMI pico, Carl Zeiss) to guarantee that no filling materials were remained. Initial working length was determined by using electronic apex locator (Raypex 5, VDW GmbH), confirmed radiographically and recorded (Figure 5).

The root canal was instrumented with K files up to 60# (Mani) and was frequently irrigated with copious 2.5% NAOCL followed by a final rinse with 5 mL 17% EDTA. Then, the root canal was dried and calcium hydroxide was placed as intracanal medicament. Then, tooth was restored with a temporary filling material (Cavit™ G, 3M ESPE). Antibiotic and analgesic on demand were prescribed. Next visit was scheduled for 2 weeks later.

**FIGURE 6:**
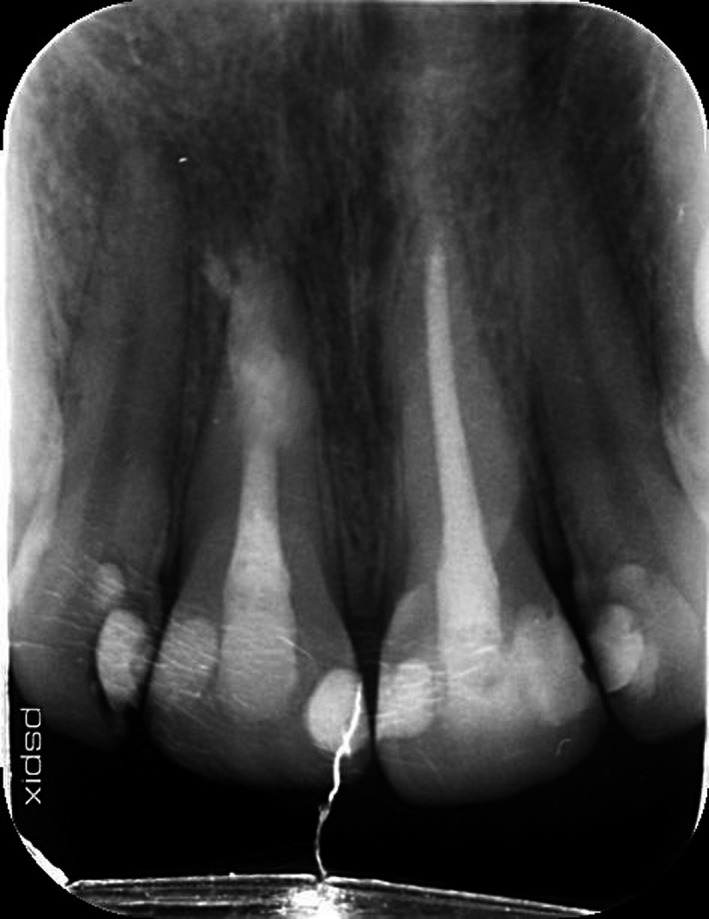
Postoperative PA of retreatment

**FIGURE 7:**
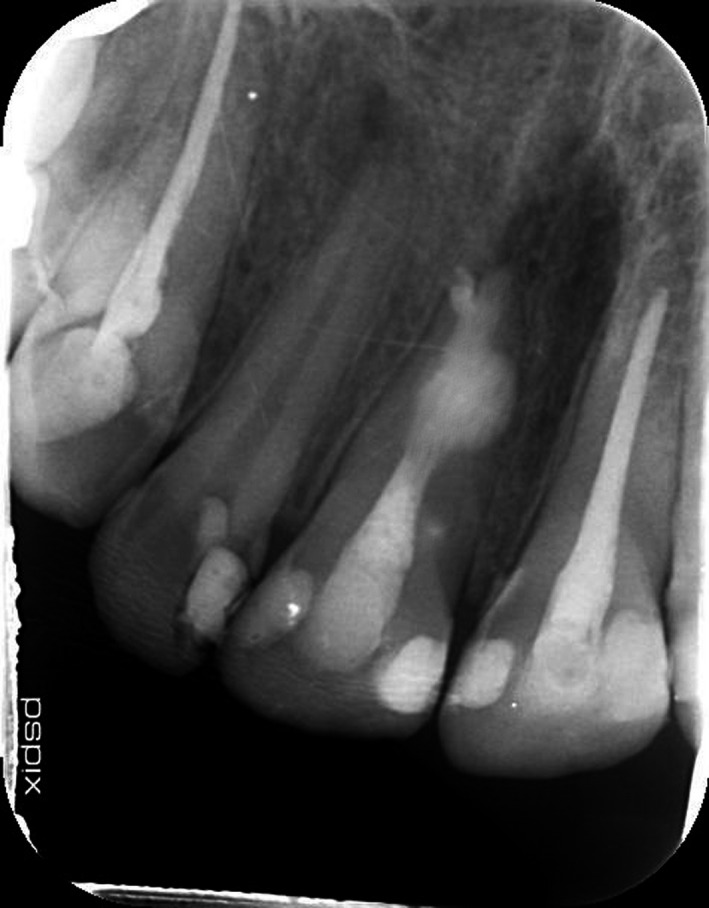
Follow‐up radiograph after 6 mo

**FIGURE 8:**
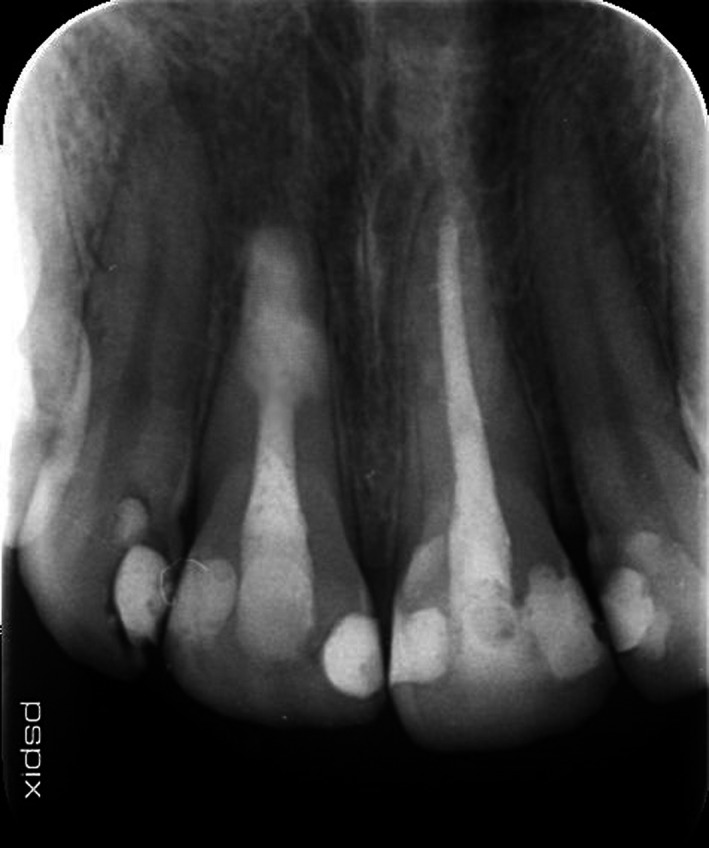
Follow‐up radiograph after 12 mo

On the subsequent visit, patient revealed no history of pain and swelling. All temporary filling material was removed, and root canal was irrigated several times with 2.5% NAOCL followed by a final rinse with 5 mL 17% EDTA and was dried by sterile paper points (Aria Dent). Biodentine was mixed according to the manufacturer's instructions. Under the dental operating microscope, Biodentine was inserted into the resorption cavity using endo gun (Medidenta) and condensed laterally against the walls of resorption cavity with root canal spreaders. The coronal third of root canal was obturated with thermoplasticized gutta‐percha (Obtura III Max, Kerr). The postoperative radiograph revealed that the external communication through the apical third of root canal was sealed accurately (Figure 6). Postobturation restoration was done by composite (3M ESPE). The patient was scheduled for appointments at 6, 12 months after treatment (Figures 7 and 8). At the recall visits, the patient was asymptomatic and the result of treatment was satisfactory.

## 3 DISCUSSION

External root resorption (ERR) is considered as one of the most important sequelae observed after common dental trauma.^4^ Early and accurate diagnosis, removal of the cause, and proper treatment of the resorbed root are imperative for successful treatment outcome.^1^ Being able to distinguish internal and external resorption is a challenge due to variations in tooth anatomy, different interpretation, and unclear radiographs.^4^ An accurate definitive diagnosis will be achieved if clinicians are expert enough to interpret radiographs correctly.^10^ There are some radiographic features of external and internal resorption can lead to correct diagnosis.^1^ In the present case, external root resorption was misdiagnosed as internal resorption primarily. However, ERR has some radiographic characteristics which make it different from IRR including apex shortened, wall of canal converges apically and canal can be followed all the way to the apex unaltered.^8^ Also, CBCT revealed the perforating external root resorption on the buccal aspect of apical third of root canal of tooth 11.

Key success of nonsurgical root canal retreatment is an effective removal of filling material and biomechanical preparation of root canal.^6^ One of the major challenges facing clinicians trying to endodontically retreat a root canal treated with the extruded gutta‐percha beyond the apical is to remove the extruded filling material.^10^ Several techniques have been introduced to remove filling materials from root canal system including H and K files, nickel titanium rotary instruments, gates glidden burs, heated instrument, ultrasonic instruments, laser, and use of adjunctive solvents.^6^ All these techniques have some advantages and disadvantages, so a dentist should be expert enough to choose one or combination of techniques with respect to quality and quantity of root filling materials and complexity of root anatomy.^11^

There is notable controversy in the literature regarding the most effective and precise technique being employed in order to remove the root filling material and preparation of apical third of roots. It has been proved that the efficiency, speed, and performance of ProTaper rotary files are higher than hand instruments to remove gutta‐percha.^12^ On the other hand, it has been shown that apical extrusion is more in rotary files than hand files.^13^ Arya et al concluded that manual instrumentation appeared superior to rotary instrumentation for the debridement in the apical third of the root canal system.^14^ Also, tactile perception helps a dentist to differentiate as hit a solid wall from passing over the apical foramen.^15^ By tactile perception, a dentist may have more control on tips of negotiating endodontic instruments in apical third of root.^16^ Saberi et al^15^ have shown using the hand instrumentation system resulted in extrusion of significantly less debris compared to the RECIPROC group and rotary instruments.

In the present case, due to overfilled original treatment, more care should be exercised in order to prevent the more extrusion of filling material beyond the apical region. So, it was decided to use the novel technique to remove the extruded gutta‐percha by wrapping H file around K file through the apical third of root. For removal of gutta‐percha from coronal and middle third of root, gates glidden and ultrasonic endodontic retreatment tips were used, respectively, as Kasam et al^17^ concluded that ultrasonic instrument was most effective to remove gutta‐percha from middle and coronal third of root canal compared to other techniques. Also, the ultrasonic instrument was used so as to better debridement and penetration of irrigation solution of hypochlorite.^13^

Biodentine is a bioactive and biocompatible calcium silicate‐based material which makes it as a favorable material to restore the resorption area.^18^ Biodentine has been introduced as a dentine substitute material; therefore, in the cases of root resorption along with the massive loss of root structure, Biodentine is the treatment of choice in order to reconstruct the damaged tissue.^8^ Biodentine has some advantages over MTA including easy handling, less setting time, and no discoloration.^18^ Biodentine does not require a two steps obturation as in the case of MTA because of its faster setting time.^8^, ^18^ In the present case, appropriate filling of resorption area with gutta‐percha and lateral condensation technique was not possible as length of the tooth was long, 23 mm, and also, the area of root resorption and perforation was located on apical third of root canal, which made it inaccessible to direct mechanical instrumentation and obturation. About the root filling, Biodentine was applied due to better consistency after mixing compared to MTA which made it possible to place in area of resorption defects with the higher adaptation.^8^ Thermoplastic gutta‐percha technique seems to give the best results when the canal walls are respected, otherwise, may be over obturated.^19^ Also, Biodentine was preferred over MTA so as to prevent the subsequent leakage between 2 sessions of treatment in the case of MTA.^18^ Furthermore, Biodentine shows apatite formation after immersion in phosphate solution indicating its bioactivity and ability to enhance the marginal sealing due to deposition of apatite structures.^8^

In the present case, Biodentine extruded beyond the apex. However, it was recommended that materials should be packed cautiously to prevent extrusion. According to some studies,^4^, ^20^ extruded MTA should not affect the outcome, similarly the present case showed that extruded Biodentine did not affect periapical healing. Besides, a year follow‐up radiograph showed no evidence of extruded Biodentine, showing dissolution of this material gradually.

## 4 CONCLUSION

In the case of orthograde retreatment of a tooth with apical extrusion of filling material, it would be better that the gutta‐percha of coronal and middle third of the root is removed by mechanical instruments including gates glidden, ultrasonic, or rotary files; however, in apical third, removal of filling material should be done by hand instrument. Also, Biodentine is the treatment of choice for filling the perforating external root resorption.

## CONFLICT OF INTEREST

Authors declare no conflict of interests.

## AUTHOR CONTRIBUTIONS

RSS: reviewed the literature, developed the concept and design of the study, and performed treatment. AJG: conceived the idea, obtained the images, and drafted the manuscript. HL: reviewed the literature, edited, and proofread the manuscript.

## Data Availability

The data used and/or analyzed in this report are available for the corresponding author [Dr Reza Sayyad Soufdoost] on responsible request.
